# Supplementary material for: Early life experiences selectively mature learning and memory abilities
Source: Nat Commun. 2020 Jan 31;11:628. doi: 10.1038/s41467-020-14461-3 (PMC6994621; doi:10.1038/s41467-020-14461-3)
Supplement: Supplementary file 1 — Supplementary Information [file 41467_2020_14461_MOESM1_ESM.pdf]

# Supplementary Information

## Early life experiences selectively mature learning and memory abilities

Bessières et al.

**Supplementary Fig. 1 (Related to Fig. 1)**

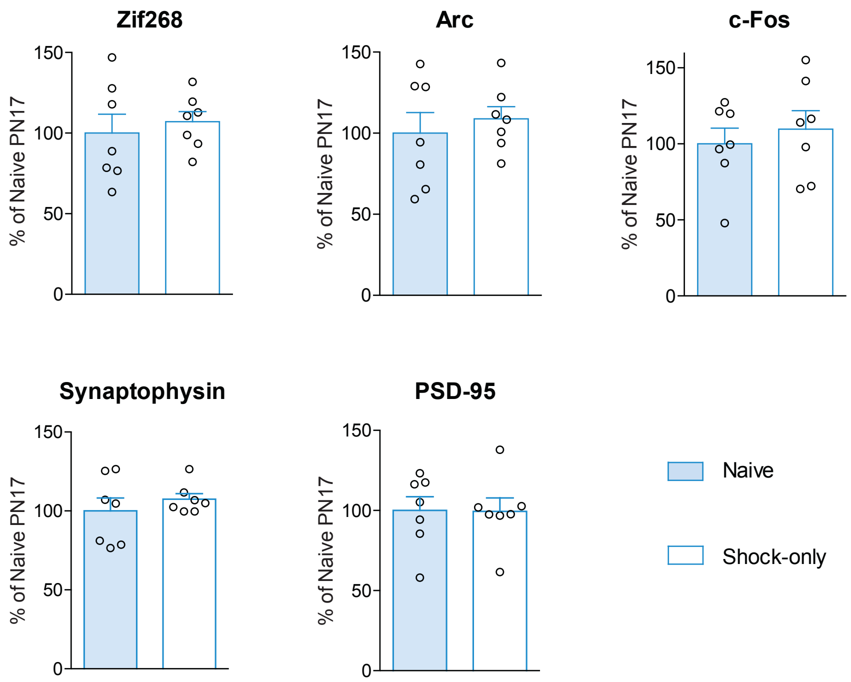


**Hippocampal molecular changes after shock-only exposure.** Densitometry of western blot analyses of whole protein extracts from rat dorsal hippocampus. Rats were euthanized 24 h after receiving an immediate footshock at PN17 (immediately after being placed on a shock grid: shock-only), and compared to rats that remained in the home cage and were euthanized at PN17 (naïve) [n=7/group, unpaired two-tailed Student's t-test, Zif268: t=0.5288, df=12, P=0.6066; Arc: t=0.5952, df=12, P=0.5628; c-Fos: t=0.6046, df=12, P=0.5567; Synaptophysin: t=0.8360, df=12, P=0.4195; PSD-95: t=0.04499, df=12, P=0.9649]. Actin was used as a loading control. Data are expressed as mean percentage ± s.e.m. of the value in the PN17 naïve group.

**Supplementary Fig. 2 (Related to Fig. 1)**

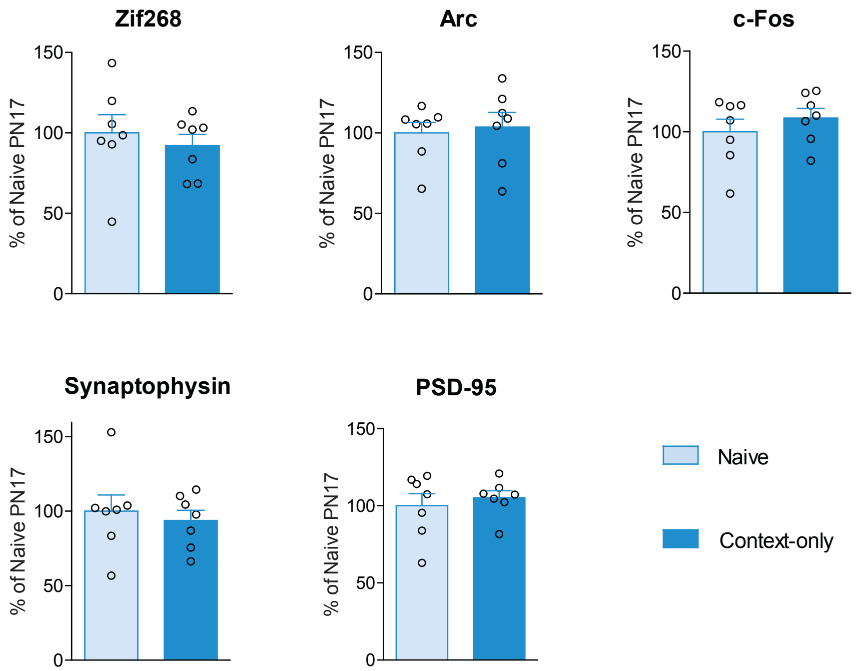


**Hippocampal molecular changes after context exposure.** Densitometry of western blot analyses of whole protein extracts from rat dorsal hippocampus. Rats were euthanized 24 h after exposure to the IA shuttlebox without footshock delivery (context-only) at PN17 [n=7/group, unpaired two-tailed Student's t-test, Zif268: t=0.5967, df=12, P=0.5618; Arc: t=0.3263, df=12, P=0.7498; c-Fos: t=0.8771, df=12, P=0.3976; Synaptophysin: t=0.4899, df=12, P=0.6331; PSD-95: t=0.5717, df=12, P=0.5781]. Actin was used as a loading control. Data are expressed as mean percentage ± s.e.m. of the value in the PN17 naïve group.

**Supplementary Fig. 3 (Related to Fig. 1)**

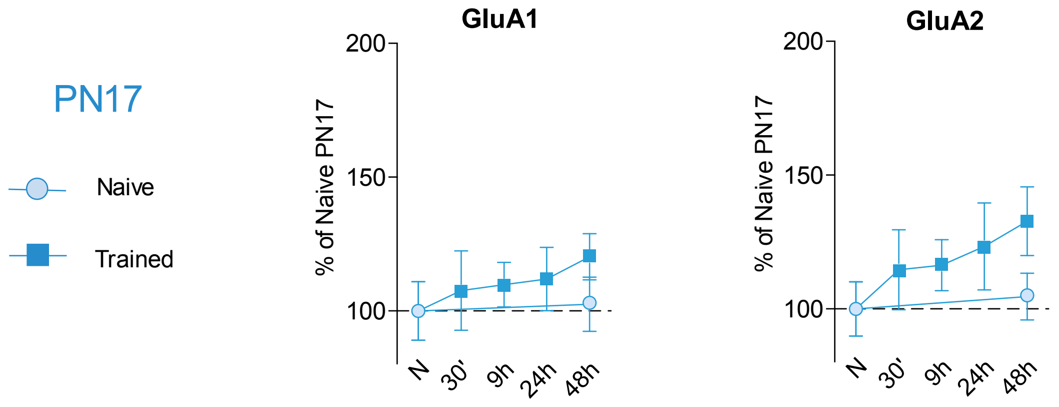


**Hippocampal levels of GluA1 and GluA2 AMPA receptor subunits following IA training.** Densitometry of GluA1 and GluA2 western blot analyses obtained from dHC whole protein extracts from rats trained in IA at PN17 and euthanized 30 min, 9h, 24h or 48h after training (naïve PN17, n=8; 30 min, n=6; 9h, n=10; 24h, n=7; 48h n=6; naïve PN19, n=6). To account for developmental differences, two groups of naïve (N) rats were used (PN17 and PN19) [One–way ANOVA followed by Dunnett's multiple comparisons test, GluA1: $F(5,37)=0.3968$, $P=0.8478$; GluA2: $F(5,37)=1.028$, $P=0.4153$]. Actin was used as a loading control. Data are expressed as mean percentage ± s.e.m. of the value in the PN17 naïve group.

## Supplementary Tables

### Supplementary Table 1 (related to Fig. 1):

| PN17 | Naïve - PN17 | 30 min | 9 h | 24 h | 48 h | Naïve – PN19 |
|---|---|---|---|---|---|---|
| **Zif268** | 100±9.9%<br>(n=8 rats) | 120.4±10.5%<br>(n=6 rats) | 130.6±8.3%<br>(n=10 rats) | 146.6±15.5%<br>(n=7 rats) | 172.1±16.9%<br>(n=6 rats) | 92.9±9.7%<br>(n=6 rats) |
| One–way ANOVA followed by Dunnett's Multiple Comparisons Test<br>$F(3,30)=3.106$, $p<0.001$<br>Student’s t-test; Naïve – PN19 *vs* 48h, $p<0.01$ | | | | | | |
| **Arc** | 100±7.6%<br>(n=8 rats) | 145.6±14.4%<br>(n=6 rats) | 179.8±10.9%<br>(n=10 rats) | 181.7±15.1%<br>(n=7 rats) | 225.6 ±33.2%<br>(n=6 rats) | 134.3±12.4%<br>(n=6 rats) |
| One–way ANOVA followed by Dunnett's Multiple Comparisons Test<br>$F(3,30)=10.78$, $p<0.0001$<br>Student’s t-test; Naïve – PN19 *vs* 48h, $p<0.05$ | | | | | | |
| **c-Fos** | 100±7.8%<br>(n=8 rats) | 94.2±5.9%<br>(n=6 rats) | 120.7±9.4%<br>(n=10 rats) | 144.8±14.1%<br>(n=7 rats) | 119.4±7.5%<br>(n=6 rats) | 118.1±11.9%<br>(n=6 rats) |
| One–way ANOVA followed by Dunnett's Multiple Comparisons Test<br>$F(3,30)=4.735$, $p=0.0088$<br>Student’s t-test; Naïve – PN19 *vs* 48h, $p>0.05$ | | | | | | |
| **pGluA1 (845)** | 100±7.5%<br>(n=6 rats) | 162.1±10.7%<br>(n=6 rats) | 220.4±11.1%<br>(n=10 rats) | 196.7±20.5%<br>(n=7 rats) | 194.3±26.3%<br>(n=6 rats) | 128.1±10.5%<br>(n=6 rats) |
| One–way ANOVA followed by Dunnett's Multiple Comparisons Test<br>$F(3, 29)=16.46$, $p<0.0001$<br>Student’s t-test; Naïve – PN19 *vs* 48h, $p<0.05$ | | | | | | |
| **pGluA1 (831)** | 100±4.5%<br>(n=6 rats) | 120.2±13.6%<br>(n=6 rats) | 134.9±14.1%<br>(n=10 rats) | 161.2±9.1%<br>(n=7 rats) | 161.9±19.1%<br>(n=6rats) | 125.0±17.8%<br>(n=6 rats) |
| One–way ANOVA followed by Dunnett's Multiple Comparisons Test<br>$F(3, 29)=4.356$, $p=0.0130$<br>Student’s t-test; Naïve – PN19 *vs* 48h, $p>0.05$ | | | | | | |
| **PSD95** | 100±17.7%<br>(n=8 rats) | 94.1±17.1%<br>(n=6 rats) | 129.9±20.8%<br>(n=9 rats) | 219.8±32.02%<br>(n=7 rats) | 258.6±61.3%<br>(n=6 rats) | 146.05±19.2%<br>(n=6 rats) |
| One–way ANOVA followed by Dunnett's Multiple Comparisons Test<br>$F(3, 30)=6.276$, $p=0.0023$<br>Student’s t-test; Naïve – PN19 *vs* 48h, $p=0.06$ | | | | | | |
| **Synapthophysin** | 100±3.8%<br>(n=8 rats) | 111.7±3.4%<br>(n=6 rats) | 140.8±10.4%<br>(n=10 rats) | 143.9±10.9%<br>(n=7 rats) | 146.1±7.8%<br>(n=6 rats) | 121.2±7.8%<br>(n= 6 rats) |
| One–way ANOVA followed by Dunnett's Multiple Comparisons Test<br>$F(3,30)=6.071$, $p=0.0027$<br>Student’s t-test; Naïve – PN19 *vs* 48h, $p<0.05$ | | | | | | |

| PN17 | Naïve – PN24 | 7 d |
|---|---|---|
| **Zif268** | 100±4.7%<br>(n=5 rats) | 116.1±11.6%<br>(n=5 rats) |
| Student’s unpaired t-test: $t=1.284$, $df=8$, $p=0.2350$ | | |
| **Arc** | 100±3.6%<br>(n=5 rats) | 102.0±2.2%<br>(n=4 rats) |

| |
|---|
| Student’s unpaired t-test: t=0.4307, df=7, p=0.6797 |

| | | |
|---|---|---|
| **c-Fos** | 100±9.01% (n=5 rats) | 98.4±4.7% (n=5 rats) |
| Student’s unpaired t-test: t=0.1540, df=8, p=0.8814 | | |
| **PSD95** | 100±4.9% (n=5 rats) | 101.9±11.8% (n=4 rats) |
| Student’s unpaired t-test: t=0.1618, df=7, p=0.8761 | | |
| **pGluA1 (845)** | 100±14.2% (n=5 rats) | 71.9±6.9% (n=5 rats) |
| Student’s unpaired t-test: t=1.773, df=8, p=0.1141 | | |
| **pGluA1 (831)** | 100±11.9% (n=5 rats) | 80.9±8.3% (n=5 rats) |
| Student’s unpaired t-test: t=1.317, df=8, p=0.2243 | | |
| **Synaptophysin** | 100±8.9% (n=5 rats) | 104.9±2.6% (n=5 rats) |
| Student’s unpaired t-test: t=0.5238, df=8, p=0.6146 | | |

| **PN24** | **Naïve – PN24** | **30 min** | **9 h** | **24 h** | **48 h** | **Naïve – PN26** |
|---|---|---|---|---|---|---|
| **Zif268** | 100±13.8% (n=4 rats) | 204.4±12.6% (n=5 rats) | 113.0±9.6% (n=6 rats) | 90.0±6.9% (n= 6 rats) | 84.3±12.3% (n=4 rats) | 92.3±10.2% (n=6 rats) |
| One–way ANOVA followed by Dunnett's Multiple Comparisons Test<br>$F(3,23)=15.1$, $p<0.001$<br>Student’s t-test;  Naïve – PN26 *vs* 48h, $p>0.05$ | | | | | | |
| **Arc** | 100±23.4% (n=4 rats) | 206.9±17.0% (n=4 rats) | 135.9±20.6% (n=6 rats) | 108.1±14.1% (n=6 rats) | 87.2±3.02% (n=4 rats) | 88.1±5.3% (n=6 rats) |
| One–way ANOVA followed by Dunnett's Multiple Comparisons Test<br>$F(3,23)=12.2$, $p<0.001$<br>Student’s t-test;  Naïve – PN26 *vs* 48h, $p>0.05$ | | | | | | |
| **c-Fos** | 100±17.9% (n=4 rats) | 177.0±13.7% (n=4 rats) | 125.3±24.2% (n=6 rats) | 106.0±12.2% (n=6 rats) | 97.4±2.9% (n=4 rats) | 104.6±3.2% (n=6 rats) |
| One–way ANOVA followed by Dunnett's Multiple Comparisons Test<br>$F(3,23)=4.489$, $p=0.0128$<br>Student’s t-test;  Naïve – PN26 *vs* 48h, $p>0.05$ | | | | | | |
| **pGluA1 (845)** | 100±8.8% (n=7 rats) | 100.1±10.8% (n=7 rats) | 109.9±13.6% (n=7 rats) | 95.8±8.3% (n=6 rats) | 87.5±8.4% (n=5 rats) | 77.5±10.2% (n=7 rats) |
| One–way ANOVA followed by Dunnett's Multiple Comparisons Test<br>$F(3,23)=0.06099$, $p=0.9798$<br>Student’s t-test;  Naïve – PN26 *vs* 48h, $p>0.05$ | | | | | | |
| **pGluA1 (831)** | 100±12.3% (n=7 rats) | 90.9±7.5% (n=7 rats) | 96.2±14.2% (n=7 rats) | 92.3±8.3% (n=7 rats) | 90.9±14.7% (n=6 rats) | 79.7±6.03% (n=7 rats) |
| One–way ANOVA followed by Dunnett's Multiple Comparisons Test<br>$F(3,23)=0.7538$, $p=0.5314$<br>Student’s t-test;  Naïve – PN26 *vs* 48h, $p>0.05$ | | | | | | |
| **Synaptophysin** | 100±8.08% (n=8 rats) | 121.6±6.6% (n=7 rats) | 120.5±6.9% (n=7 rats) | 106.6±10.3% (n=7 rats) | 101.4±10.1% (n=6 rats) | 108.6±5.1% (n=7 rats) |

| | | | | | | |
|---|---|---|---|---|---|---|
| One–way ANOVA followed by Dunnett's Multiple Comparisons Test<br>$F(3,23)=0.08686$, $p=0.9665$<br>Student's t-test; Naïve – PN26 *vs* 48h, $p>0.05$ | | | | | | |
| **PSD95** | 100±11.3%<br>(n=7 rats) | 102.8±6.5%<br>(n=7 rats) | 96.2±9.3%<br>(n=7 rats) | 89.9±11.4%<br>(n=7 rats) | 92.6±11.4%<br>(n=6 rats) | 88.8±5.6%<br>(n=7 rats) |
| One–way ANOVA followed by Dunnett's Multiple Comparisons Test<br>$F(3,23)=0.2067$, $p=0.8907$<br>Student's t-test; Naïve – PN19 *vs* 48h, $p>0.05$ | | | | | | |

## Supplementary Table 2 (related to Fig. 2a):

| Fig. 2a | Naïve | Tr-IgG | Tr-Anti-BDNF |
|---|---|---|---|
| **Synaptophysin** | 100±18.7 %<br>(n=8 rats) | 224.6± 18.0%<br>(n=6 rats) | 138.3±22.7%<br>(n=7 rats) |
| One–way ANOVA followed by Tukey's Multiple Comparisons Test<br>$F(2,18)=9.57$, $p=0.0015$ | | | |
| **PSD95** | 100±12.07%<br>(n=7 rats) | 237.4±41.6%<br>(n=6 rats) | 133.9±21.7%<br>(n=5 rats) |
| One–way ANOVA followed by Tukey's Multiple Comparisons Test<br>$F(2,16)=7.59$, $p=0.0049$ | | | |
| **pGluA (845)** | 100±1.4%<br>(n=4 rats) | 157.9±18.7%<br>(n=6 rats) | 146.1±24.2%<br>(n=6 rats) |
| One–way ANOVA followed by Tukey's Multiple Comparisons Test<br>$F(2,17)=4.51$, $p=0.0269$ | | | |
| **pGluA (831)** | 100±8.9%<br>(n=7 rats) | 149.7±17.5%<br>(n=4 rats) | 113.3±8.3%<br>(n=6 rats) |
| One–way ANOVA followed by Tukey's Multiple Comparisons Test<br>$F(2,17)=5.319$, $p=0.0161$ | | | |

## Supplementary Table 3 (related to Fig. 2b):

| Fig. 2b | N-SCR | Tr-SCR | Tr-AS |
|---|---|---|---|
| **PSD95** | 100±6.4%<br>(n=4 rats) | 183.5±19.1%<br>(n=4 rats) | 87.9±7.9%<br>(n=4 rats) |
| One–way ANOVA followed by Tukey's Multiple Comparisons Test<br>$F(2,9)=17.33$, $P=0.0008$ | | | |

## Supplementary Table 4 (related to Fig. 2c,d):

| Fig. 2c | Mean Latency (s) | | | |
|---|---|---|---|---|
| **PN17** | **Acq** | **Test 1 (T1)** | **T2/Tr** | **Test 3 (T3)** |
| PN17 SCR (n=7) | 31.4±15.9 | 37.8±12.2 | 606.7±74.7 | - |
| PN17 AS (n=8) | 37.2±15.8 | 43.6±14.9 | 93.2±42.3 | 821.7±48.4 |

| |
|---|
| Two–way RM ANOVA followed by Bonferroni's Multiple Comparisons Test<br>Interaction: F (2, 26) = 32.3627, P < 0.0001<br>Time: F (2, 26) = 46.8857, P < 0.0001<br>Treatment: F (1, 13) = 37.5766, P < 0.0001<br><br>Student's paired t-test T2 vs. T3<br>t=13.28, df=7, P<0.0001 |

| **Fig. 2d** | **Mean Latency (s)** | | |
|---|---|---|---|
| **PN24** | **Acq** | **Test 1 (T1)** | **Test 2 (T2)** |
| PN24 SCR (n=7) | 16.8±4.4 | 577.6±128.8 | 701.3±121.5 |
| PN24 AS (n=8) | 14.5±3.6 | 689.1±58.9 | 830.0±37.4 |
| Two–way RM ANOVA followed by Bonferroni's Multiple Comparisons Test<br>Interaction: F (2, 26) = 0.804744, P = 0.4580<br>Time: F (2, 26) = 101.758, P < 0.0001<br>Treatment: F (1, 13) = 0.934685, P = 0.3513 | | | |

## Supplementary Table 5 (related to Fig. 3a-d):

| **Fig. 3a** | **RMP (mV)** | |
|---|---|---|
| | Naive | Trained |
| PN17 (n=14) | -58.2±1.1 | -61.2±0.6 |
| PN24 (n=14) | -60.6±0.9 | -60.6±0.6 |
| Two–way ANOVA followed by Bonferroni's Multiple Comparisons Test<br>Interaction: F (1, 52) = 3.07420, P = 0.0854<br>Age: F (1, 52) = 1.16209, P = 0.2860<br>Condition: F (1, 52) = 3.21188, P = 0.0789 | | |

| **Fig. 3a** | **Rin (mΩ)** | |
|---|---|---|
| | Naive | Trained |
| PN17 (n=14) | 137.1±8.8 | 156.5±9.6 |
| PN24 (n=14) | 160.6±15.7 | 181.8±15.3 |
| Two–way ANOVA followed by Bonferroni's Multiple Comparisons Test<br>Interaction: F (1, 52) = 0.0046351, P = 0.9460<br>Age: F (1, 52) = 3.64904, P = 0.0616<br>Condition: F (1, 52) = 2.54592, P = 0.1166 | | |

| **Fig. 3b** | **AMPA amplitude (mV)** | |
|---|---|---|
| | Naive | Trained |
| PN17 (n=14) | 6.7±0.9 | 13.4±1.5 |
| PN24 (n=14) | 9.6±1.2 | 8.8±1.1 |
| Two–way ANOVA followed by Bonferroni's Multiple Comparisons Test<br>Interaction: F (1, 52) = 9.70420, P = 0.0030<br>Age: F (1, 52) = 0.529193, P = 0.4702<br>Condition: F (1, 52) = 6.01344, P = 0.0176 | | |

| **Fig. 3c** | **AMPA decay (ms)** | |
|---|---|---|
| | Naive | Trained |

| | | |
|---|---|---|
| PN17 (n=14) | 104.9±15.5 | 56.4±4.2 |
| PN24 (n=14) | 76.9±3.2 | 65.5±6.9 |
| Two–way ANOVA followed by Bonferroni's Multiple Comparisons Test<br>Interaction: F (1, 52) = 4.36921, P = 0.0415<br>Age: F (1, 52) = 1.12068, P = 0.2947<br>Condition: F (1, 52) = 11.4110, P = 0.0014 | | |

| Fig. 3d | ISI 50 | |
|---|---|---|
| | Naive | Trained |
| PN17 (n=14) | 1.51±0.11 | 1.40±0.12 |
| PN24 (n=14) | 1.27±0.19 | 1.01±0.10 |
| Two–way ANOVA followed by Bonferroni's Multiple Comparisons Test<br>Interaction: F (1, 52) = 0.324764, P = 0.5720<br>Age: F (1, 52) = 5.28333, P = 0.0270<br>Condition: F (1, 52) = 1.86005, P = 0.1804 | | |

| Fig. 3d | ISI 200 | |
|---|---|---|
| | Naive | Trained |
| PN17 (n=14) | 1.25±0.09 | 1.42±0.08 |
| PN24 (n=14) | 1.05±0.04 | 0.99±0.04 |
| Two–way ANOVA followed by Bonferroni's Multiple Comparisons Test<br>Interaction: F (1, 39) = 1.90058, P = 0.1759<br>Age: F (1, 39) = 14.3378, P = 0.0005<br>Condition: F (1, 39) = 0.403775, P = 0.5289 | | |

## Supplementary Table 6 (related to Fig. 3e-h):

| Fig. 3e | N-SCR (n=23) | Tr-SCR (n=19) | Tr-AS (n=18) |
|---|---|---|---|
| **RMP (mV)** | -58.1±0.8 | -58.8±0.9 | -58.9± 0.9 |
| One–way ANOVA followed by Tukey's Multiple Comparisons Test<br>F(2,57) = 0.2644, P = 0.7686 | | | |

| Fig. 3e | N-SCR (n=23) | Tr-SCR (n=19) | Tr-AS (n=18) |
|---|---|---|---|
| **Rin (mΩ)** | 186.7±10.2 | 178.1±11.3 | 165.2±11.6 |
| One–way ANOVA followed by Tukey's Multiple Comparisons Test<br>F(2,57) = 0.9706, P = 0.3850 | | | |

| Fig. 3f | N-SCR (n=23) | Tr-SCR (n=19) | Tr-AS (n=18) |
|---|---|---|---|
| **AMPA amplitude (mV)** | 6.95±0.9 | 14.96±1.02 | 8.6±1.05 |

| |
|---|
| One–way ANOVA followed by Tukey's Multiple Comparisons Test<br>F(2,57) = 18.1696, P < 0.0001 |

| Fig. 3g | N-SCR (n=23) | Tr-SCR (n=19) | Tr-AS (n=18) |
|---|---|---|---|
| **AMPA decay (ms)** | 91.3±5.5 | 68.5±6.1 | 93.6±6.3 |
| One–way ANOVA followed by Tukey's Multiple Comparisons Test<br>F(2,57) = 5.2551, P = 0.008 | | | |

| Fig. 3h | N-SCR (n=23) | Tr-SCR (n=19) | Tr-AS (n=18) |
|---|---|---|---|
| **ISI 50** | 1.37±0.03 | 1.37±0.04 | 1.36±0.04 |
| One–way ANOVA followed by Tukey's Multiple Comparisons Test<br>F(2,57) = 0.0270, P = 0.9733 | | | |

| Fig. 3h | N-SCR (n=23) | Tr-SCR (n=19) | Tr-AS (n=18) |
|---|---|---|---|
| **ISI 200** | 1.22±0.02 | 1.18±0.03 | 1.22±0.03 |
| One–way ANOVA followed by Tukey's Multiple Comparisons Test<br>F(2,57) = 0.7177, P = 0.4922 | | | |

## Supplementary Table 7 (related to Fig. 4):

| Fig. 4a | Mean Latency (s) | | | |
|---|---|---|---|---|
| **PN17** | **Tr1** | **Tr2** | **T1** | **T2** |
| PN17 Naive (n=5) | - | - | 20.0±7.4 | 14.3±4.9 |
| PN17 shock-only (n=6) | - | - | 24.3±7.3 | 30.6±16.9 |
| PN17 + PN18 (n=9) | 14.4±8.6 | 42.3±15.5 | 751.4±56.6 | 834.6±22.9 |
| Two–way RM ANOVA followed by Bonferroni's Multiple Comparisons Test<br>Interaction: F (2, 17) = 1.24724, P = 0.3123<br>Time: F (1, 17) = 1.07976, P = 0.3133<br>Condition: F (2, 17) = 303.143, P < 0.0001 | | | | |

| Fig. 4b | Mean Latency (s) | | | |
|---|---|---|---|---|
| **PN17** | **Tr1** | **Tr2** | **T1** | **T2** |
| PN17 (n=6) | 37.6±7.1 | - | 34.2±8.2 | 21.6±6.5 |
| PN19 (n=8) | 13.2±2.5 | - | 75.3±13.03 | 49.02±9.4 |
| PN17 + PN19 (n=8) | 18.3±5.5 | 39.6±26.7 | 639.5±48.2 | 603.5±58.01 |
| Two–way RM ANOVA followed by Bonferroni's Multiple Comparisons Test<br>Interaction: F (6, 42) = 70.8959 P < 0.0001<br>Time: F (3, 21) = 62.0631 P < 0.0001<br>Condition: F (2, 14) = 292.637 P < 0.0001 | | | | |

| Fig. 4c | Mean Latency (s) | | | |
|---|---|---|---|---|
| PN17 | Tr1 | Tr2 | T1 | T2 |
| 2xPN17 (n=6) | 14.6±7.9 | 69.6±25.2 | 186.2±54.6 | 249.04±110.4 |
| 2xPN18 (n=7) | 11.9±3.08 | 26.5±7.6 | 160.9±59.7 | 300.9±60.9 |
| PN17 + PN18 (n=9) | 27.4±8.4 | 56.2±19.9 | 737.9±58.08 | 878.8±10.5 |
| Two–way RM ANOVA followed by Bonferroni's Multiple Comparisons Test<br>Interaction: F (6, 57) = 22.2596, P < 0.0001<br>Time: F (3, 57) = 84.0132, P < 0.0001<br>Condition: F (2, 19) = 56.1766, P < 0.0001 | | | | |

| Fig. 4d | Mean Latency (s) | | |
|---|---|---|---|
| | Tr1 | Tr2 | T1 |
| PN17 (n=10) | 30.0±7.5 | 45.8±10.1 | 220.7±48.6 |
| One–way RM ANOVA followed by Tukey's Multiple Comparisons Test<br>F (2, 27) = 0.7010, P = 0.0053 | | | |

| Fig. 4e | Mean Latency (s) | | | | |
|---|---|---|---|---|---|
| PN17 | Tr1 | Tr2 | T1 | T2/Tr | T3 |
| PN17 SCR (n=6) | 24.8±4.3 | 20.1±6.08 | 498.8±75.2 | 577.1±84.8 | - |
| PN17 AS (n=6) | 29.8±6.2 | 32.8±9.3 | 127.9±51.7 | 79.4±30.5 | 752.8±57.2 |
| Two–way RM ANOVA followed by Bonferroni's Multiple Comparisons Test<br>Interaction: F (3, 30) = 17.0618, P < 0.0001<br>Time: F (3, 30) = 28.8797, P < 0.0001<br>Treatment: F (1, 10) = 38.8975, P < 0.0001 | | | | | |

## Supplementary Table 8 (related to Fig. 5a,b):

| | Mean Latency (s) | | | | | |
|---|---|---|---|---|---|---|
| Fig. 5a | Tr1 | Tr2 | T1 | T2 | T3 | T4 |
| PN17 (n=8) | 9.5±2.6 | 40.2±15.1 | 683.6±59.5 | 26.9±5.8 | 714.2±96.8 | 23.4±6.6 |
| One–way RM ANOVA followed by Tukey's Multiple Comparisons Test<br>F(5,47)= 51.57, P<0.0001 | | | | | | |
| Fig. 5b | Tr1 | Tr2 | T1 | T2 | T3 | T4 |
| PN17 (n=7) | 14.6±3.7 | 22.2±9.18 | 710.8±106.7 | 675.6±56.9 | 723.8±71.01 | 714.6±71.9 |
| One–way RM ANOVA followed by Tukey's Multiple Comparisons Test<br>F(5,41)= 26.72, P<0.0001 | | | | | | |

## Supplementary Table 9 (related to Fig. 5c):

| Fig. 5c | % Preference | | |
|---|---|---|---|
| | Tr1 | Tr2 | Test |
| PN17 (n=8) | 52.4±2.9 | - | 50.7±3.1 |
| PN17A + PN18A (n=8) | 50.6±2.1 | 50.8±3.7 | 67.4±3.4 |
| PN17A + PN18B (n=8) | 47.4±2.1 | 46.2±5.1 | 72.5±3.1 |

PN17: Student's paired t-test
t=0.6020, df=7, P=0.5662

PN17A + PN18A: One–way RM ANOVA followed by Tukey's Multiple Comparisons Test
F (2,23) = 11.34, P = 0.0035

PN17A + PN18A: One–way RM ANOVA followed by Tukey's Multiple Comparisons Test
F (2,23) = 15.61, P = 0.0012

## Supplementary Table 10 (related to Fig. 5d):

| Fig. 5d (IA) | Mean Latency (s) | | |
|---|---|---|---|
| | Acq | T1 | T2 |
| PN17 (n=8) | 16.5±3.6 | 32.3±7.1 | 621.8±71.2 |

One–way RM ANOVA followed by Tukey's Multiple Comparisons Test
F(2,23)= 66.58, P<0.0001

| Fig. 5d (nOL) | % Preference | |
|---|---|---|
| | Tr | Test |
| PN17 (n=8) | 52.9±6.5 | 49.1±3.4 |

Student's paired t-test
t=0.4561, df=7, P=0.6622

## Supplementary Table 11 (related to Fig. 5e):

| Fig. 5e (nOL) | % Preference | |
|---|---|---|
| | Tr | Test |
| Veh (n=7) | 49.7±6.4 | 52.6±2.03 |
| BDNF (n=7) | 46.1±8.1 | 76.3±3.5 |

Two–way RM ANOVA followed by Bonferroni's Multiple Comparisons Test
Interaction: F (1, 12) = 6.38547, P = 0.0266
Time: F (1, 12) = 9.36432, P = 0.0099
Treatment: F (1, 12) = 3.18180, P = 0.0998

| Fig. 5e (IA) | Mean Latency (s) | | | |
|---|---|---|---|---|
| PN17 | Acq | T1 | T2 | T3 |
| Veh (n=7) | 12.7±3.9 | 25.4±5.5 | 46.4±13.5 | 606.5±78.0 |
| BDNF (n=7) | 24.8±5.3 | 36.3±10.3 | 51.9±13.3 | 696.7±81.9 |

| |
|---|
| Two–way RM ANOVA followed by Bonferroni's Multiple Comparisons Test<br>Interaction: $F(3, 36) = 0.480926$, $P = 0.6976$<br>Time: $F(3, 36) = 112.788$, $P < 0.0001$<br>Treatment: $F(1, 12) = 1.12432$, $P = 0.3099$ |

## Supplementary Table 12 (related to Fig. 6a):

| **Fig. 6a** | N-SCR | Tr-SCR | Tr-AS |
|---|---|---|---|
| **c-Fos** | 100±2.4%<br>(n=6 rats) | 156.6±7.9%<br>(n=6 rats) | 109.6±7.6%<br>(n=6 rats) |
| One–way ANOVA followed by Tukey's Multiple Comparisons Test<br>$F(2, 15) = 21.92, P < 0.0001$ | | | |

## Supplementary Table 13 (related to Fig. 6b):

| **Fig. 6b** | **Mean Latency (s)** | | | | |
|---|---|---|---|---|---|
| **PN17** | **Tr1** | **Tr2** | **T1** | **T2/Tr** | **T3** |
| PN17 SCR (n=8) | 29.6±7.3 | 46.1±9.6 | 490.1±80.8 | 485.7±51.5 | - |
| PN17 AS (n=8) | 26.5±4.2 | 25.6±5.9 | 84.3±32.05 | 35.9±9.5 | 709.9±48.7 |
| Two–way RM ANOVA followed by Bonferroni's Multiple Comparisons Test<br>Interaction: $F(3, 28) = 81.9781$ $P < 0.0001$<br>Time: $F(3, 28) = 36.1490$ $P < 0.0001$<br>Treatment: $F(1, 7) = 5.40951$ $P < 0.0001$ | | | | | |

## Supplementary Table 14 (related to Fig. 7a):

| **Fig. 7a** | **% Freezing** | | |
|---|---|---|---|
| | **Tr** | **T1** | **T2** |
| PN17 (n=9) | 8.3±2.6 | 64.3±5.3 | 5.9±1.9 |
| One–way RM ANOVA followed by Tukey's Multiple Comparisons Test: $F(2,26) = 78.20, P<0.0001$ | | | |

## Supplementary Table 15 (related to Fig. 7b):

| **PN17** | **Naïve - PN17** | **30 min** | **24 h** | **48 h** | **Naïve – PN19** |
|---|---|---|---|---|---|
| **c-Fos** | 100±2.2%<br>(n=5 rats) | 121.7±6.3%<br>(n=5 rats) | 151.6±11.6%<br>(n=6 rats) | 101.6±11.7%<br>(n=5 rats) | 91.8±7.7%<br>(n=3 rats) |
| One–way ANOVA followed by Dunnett's Multiple Comparisons Test<br>$F(3, 17) = 7.128, P = 0.0026$<br>Student's t-test; Naïve – PN19 *vs* 48h: $t=0.5938, df=6, P=0.5743$ | | | | | |

## Supplementary Table 16 (related to Fig. 7c):

| Fig. 7c | % Freezing | | |
|---|---|---|---|
| | Tr | T1 | T2 |
| cFOS/DREADD + CNO (n=5) | 12.4±2.3 | 40.8±5.02 | 31.9±9.1 |
| Control + CNO (n=5) | 8.3±4.9 | 7.6±2.8 | 4.2±2.0 |
| Two–way RM ANOVA followed by Bonferroni's Multiple Comparisons Test<br>Interaction: $F (2, 16) = 3.56753$, $P = 0.0523$<br>Time: $F (2, 16) = 2.84896$, $P = 0.0874$<br>Condition: $F (1, 8) = 98.6686$, $P < 0.0001$ | | | |

## Supplementary Table 17 (related to Fig. 7d):

| Fig. 7d | % Freezing | | | |
|---|---|---|---|---|
| | Tr | T1 | T2/Tr | T3 |
| PN17 + PN24 (n=6) | 11.2±3.8 | 62.2±10.5 | 16.8±4.9 | 45.3±9.1 |
| Control (n=6) | - | - | 10.7±3.5 | 20.9±8.4 |
| Two–way RM ANOVA followed by Bonferroni's Multiple Comparisons Test<br>Interaction: $F (3, 30) = 6.59479$ $P = 0.0015$<br>Time: $F (3, 30) = 7.36323$ $P = 0.0008$<br>Condition: $F (1, 10) = 131.064$ $P < 0.0001$ | | | | |

## Supplementary Table 18 (related to Fig. 8a):

| Fig. 7e | % Freezing | | | |
|---|---|---|---|---|
| | Tr1 | Tr2 | T1 | T2 |
| PN17+ PN18 (n=6) | 6.3±2.3 | 56.5±3.5 | 59.9±3.6 | 25.4±1.8 |
| PN17 (n=6) | 4.2±1.6 | - | 8.5±2.6 | 1.4±0.6 |
| Two–way RM ANOVA followed by Bonferroni's Multiple Comparisons Test<br>Interaction: $F (3, 30) = 73.3626$ $P < 0.0001$<br>Time: $F (3, 30) = 80.8402$ $P < 0.0001$<br>Condition: $F (1, 10) = 271.944$ $P < 0.0001$ | | | | |

## Supplementary Table 19 (related to Fig. 8b):

| Fig. 7f | % Preference | |
|---|---|---|
| | Tr | Test |
| PN17 - 1 min (n=9) | 50.7±2.04 | 75.3±2.4 |
| PN17 - 2 h (n=9) | 48.6±2.1 | 48.05±2.7 |
| PN17 - 1 min: Student's paired t-test<br>$t=6.855$, $df=8$, $P < 0.0001$<br><br>PN17 – 2 h: Student's paired t-test | | |

| |
|---|
| t=0.1152, df=8, P = 0.9111 |

## Supplementary Table 20 (related to Fig. 8c):

| **Fig. 7g (nOL)** | **% Preference** | |
|---|---|---|
| | **Tr** | **Test (1min)** |
| PN17 - 1 min (n=8) | 50.4±3.5 | 69.4±3.6 |
| Student's paired t-test<br>t=4.958, df=7, P = 0.0016 | | |

| **Fig. 7g (CFC)** | **% Freezing** | | |
|---|---|---|---|
| | **Tr** | **T1** | **T2** |
| PN17 (n=8) | 4.1±0.5 | 70.6±4.9 | 11.2±2.9 |
| One–way RM ANOVA followed by Tukey's Multiple Comparisons Test<br>F(2,23)= 106.3, P<0.0001 | | | |

## Supplementary Table 21 (related to Fig. 8d):

| **Fig. 7h** | **% Freezing** | | |
|---|---|---|---|
| **CFC** | **Tr** | **T1** | **T2** |
| cFos/DREADD + CNO (n=4) | 8.9±1.9 | 48.9±5.9 | 33.3±8.6 |
| Control + CNO (n=4) | 8.1±2.4 | 12.7±2.7 | 7.5±6.4 |
| Two–way RM ANOVA followed by Bonferroni's Multiple Comparisons Test<br>Interaction: F (2, 12) = 5.29795, P = 0.0224<br>Time: F (2, 12) = 7.90883, P = 0.0064<br>Condition: F (1, 6) = 30.9896, P = 0.0014 | | | |
| **Fig. 7h** | **% Preference** | | |
| **nOL** | **Tr** | **Test** | |
| cFOS/DREADD + CNO (n=4) | 46.5±2.8 | 56.2±2.9 | |
| Control + CNO (n=4) | 41.3±3.9 | 52.8±3.8 | |
| Two–way RM ANOVA followed by Bonferroni's Multiple Comparisons Test<br>Interaction: F (1, 6) = 0.0437526, P = 0.8412<br>Time: F (1, 6) = 6.43775, P = 0.0442<br>Condition: F (1, 6) = 3.34748, P = 0.1171 | | | |
