# Supplementary material for: Early life experiences selectively mature learning and memory abilities
Source: Nat Commun. 2020 Jan 31;11:628. doi: 10.1038/s41467-020-14461-3 (PMC6994621; doi:10.1038/s41467-020-14461-3)
Supplement: Supplementary file 3 — Reporting Summary [file 41467_2020_14461_MOESM3_ESM.pdf]

nature research

Corresponding author(s): Cristina M. Alberini

Last updated by author(s): Dec 23, 2019

Reporting Summary

Nature Research wishes to improve the reproducibility of the work that we publish. This form provides structure for consistency and transparency in reporting. For further information on Nature Research policies, see Authors & Referees and the Editorial Policy Checklist .

Statistics

For all statistical analyses, confirm that the following items are present in the figure legend, table legend, main text, or Methods section.

n/a	Confirmed	
☐	☒	The exact sample size (n) for each experimental group/condition, given as a discrete number and unit of measurement
☐	☒	A statement on whether measurements were taken from distinct samples or whether the same sample was measured repeatedly
☐	☒	The statistical test(s) used AND whether they are one- or two-sided<br>*Only common tests should be described solely by name; describe more complex techniques in the Methods section.*
☒	☐	A description of all covariates tested
☐	☒	A description of any assumptions or corrections, such as tests of normality and adjustment for multiple comparisons
☐	☒	A full description of the statistical parameters including central tendency (e.g. means) or other basic estimates (e.g. regression coefficient) AND variation (e.g. standard deviation) or associated estimates of uncertainty (e.g. confidence intervals)
☐	☒	For null hypothesis testing, the test statistic (e.g. F, t, r) with confidence intervals, effect sizes, degrees of freedom and P value noted<br>*Give P values as exact values whenever suitable.*
☒	☐	For Bayesian analysis, information on the choice of priors and Markov chain Monte Carlo settings
☒	☐	For hierarchical and complex designs, identification of the appropriate level for tests and full reporting of outcomes
☒	☐	Estimates of effect sizes (e.g. Cohen's d, Pearson's r), indicating how they were calculated

Our web collection on statistics for biologists contains articles on many of the points above.

Software and code

Policy information about availability of computer code

Data collection	Odyssey Image Studio Lite (LI-COR Biosciences), Ethovison XT (Noldus)
Data analysis	Prism 7 GraphPad, Image Studio Lite (LI-COR Biosciences)

For manuscripts utilizing custom algorithms or software that are central to the research but not yet described in published literature, software must be made available to editors/reviewers. We strongly encourage code deposition in a community repository (e.g. GitHub). See the Nature Research guidelines for submitting code & software for further information.

Data

Policy information about availability of data

All manuscripts must include a data availability statement. This statement should provide the following information, where applicable:

- Accession codes, unique identifiers, or web links for publicly available datasets
- A list of figures that have associated raw data
- A description of any restrictions on data availability

The data that support the findings of this study are available from the corresponding author upon reasonable request. The source data underlying Figs 1-7 and Supplementary Figs 1-3 are provided as a Source Data file.

Field-specific reporting

Please select the one below that is the best fit for your research. If you are not sure, read the appropriate sections before making your selection.

☒ Life sciences ☐ Behavioural & social sciences ☐ Ecological, evolutionary & environmental sciences

Life sciences study design

All studies must disclose on these points even when the disclosure is negative.

Sample size	No statistical methods were used to predetermine sample size. Sample size used is based on common and accepted size in the field.
Data exclusions	Rats with incorrect guide-cannulae placement were excluded from the study (less than 5%).
Replication	Results reported are based on at least 2 replicated experiments, except for the data depicted in Fig. 7h, which were obtained after one single experiment. All replications were successful.
Randomization	No randomization was used to collect the data.
Blinding	Behavioral, biochemical and electrophysiological data collection and analysis were performed blind to the conditions of the experiments.

Reporting for specific materials, systems and methods

We require information from authors about some types of materials, experimental systems and methods used in many studies. Here, indicate whether each material, system or method listed is relevant to your study. If you are not sure if a list item applies to your research, read the appropriate section before selecting a response.

Materials & experimental systems

n/a	Involved in the study	
☐	☒	Antibodies
☒	☐	Eukaryotic cell lines
☒	☐	Palaeontology
☐	☒	Animals and other organisms
☒	☐	Human research participants
☒	☐	Clinical data

Methods

n/a	Involved in the study	
☒	☐	ChIP-seq
☒	☐	Flow cytometry
☒	☐	MRI-based neuroimaging

Antibodies

Antibodies used	Primary antibodies used at the indicated dilutions: anti-Arc (1:10000, Synaptic System, cat# 156 003), anti-c-Fos (1:200, Millipore; cat# PC05), anti-Zif268 (1:1000, Cell Signaling Technology, cat#4153S), anti-Synaptophysin (1:1000, Cell Signaling Technology, cat# 5467), anti-PSD95 (1:1000, Cell Signaling Technology, cat# 2507S), anti-pAMPA Receptor GluA1 (Ser845) (1:1000, Cell Signaling Technology, cat# 8084S), anti-pAMPA Receptor GluA1 (Ser831) (1:1000, Abcam, cat# ab109464), anti-Actin (1:20,000, Santa Cruz Biotechnology, cat# sc-47778)<br>Secondary antibodies: goat anti-mouse IRDye 680LT (1:10,000, cat# 926-68020) and goat anti-rabbit IRDye 800CW (1:10,000, cat# 926-32211) from LI-COR Biosciences.
Validation	The antibodies used in this study were commercially available and had been validated in previous publications as indicated on the website of each manufacturer.

Animals and other organisms

Policy information about studies involving animals; ARRIVE guidelines recommended for reporting animal research

Laboratory animals	Rats: Male and Female PN17 and PN24 Long Evans rats, Charles River Laboratories, cat# 2308852.<br>Mice: Male and female PN17 cfos-htTA/tetO-hM3Dq mice were obtained by crossing B6.Cg-Tg(Fos-tTA,Fos-EGFP*)1Mmay/J mice (The Jackson Laboratory, cat# 018306) and Tg(tetO-CHRM3*)1Blr/J (The Jackson Laboratory, cat# 014093).
Wild animals	No wild animal was used.
Field-collected samples	No field-collected sample was used.
Ethics oversight	All procedures complied with the US National Institute of Health Guide for the Care and Use of Laboratory Animals and were approved by the New York University Animals Care Committees.

Note that full information on the approval of the study protocol must also be provided in the manuscript.
